# Nutritional Challenges in Hemodialysis: A Perspective from Albania

**DOI:** 10.5152/eurasianjmed.2026.251196

**Published:** 2026-06-17

**Authors:** Paola Pina, Elona Prifti, Fjona Nasto, Mikel Shella, Xhoela Shtӫmbari, Senada Kaca

**Affiliations:** 1 Department of Nursing, Aleksandër Xhuvani University of Elbasan Faculty of Medical Technical Sciences, Elbasan, Albania; 2 Department of Nephrology and Dialysis, American Hospital, Tiranӫ, Albania; 3 Department of Nephrology, Elbasan Regional Hospital, Elbasan, Albania; 4 Department of Family Medicine, Polyclinic Nr.1, Lushnje, Albania; 5 Department of Pediatrics, Shkodӫr Regional Hospital, Shkodӫr, Albania

**Keywords:** Fluid management, hemodialysis patients, malnutrition

## Abstract

Advanced kidney disease and renal replacement therapy are associated with numerous metabolic and nutritional imbalances. Nutrition plays a major role in reducing complications and improving quality of life. The aim of this structured narrative review was to explore the specific nutritional and fluid management challenges among hemodialysis patients in Albania. A search of various databases (PubMed, Cochrane Library, Medscape, UpToDate, and Google Scholar) was conducted for publications from 2000 to 2025, identifying 49 studies for inclusion. Evidence from the literature indicates that patients undergoing hemodialysis present with multiple vitamin and mineral deficiencies. Common issues include vitamin C, vitamin D, B6, B12, carnitine deficiency, and malnutrition prevalence of 43%-61% across various studies. In Albania, care is constrained by low household incomes; traditional diets high in phosphorus, potassium, and salt; the absence of renal dietitians; inconsistent patient and family education; and excessive interdialytic weight gain. Recommendations emphasize the need to implement routine measurements of biochemical markers such as serum prealbumin, anthropometric measurements such as body mass index and mid-upper arm circumference, evaluation of dietary intake at least every 3 months and objective monitoring tools such as bioimpedance spectroscopy for assessing extracellular volume status. The lack of official data on the nutritional status of hemodialysis patients in Albania highlights the urgent need for multicenter, collaborative research, and the establishment of improved, evidence-based nutritional regimens. Implementing these clinical and policy measures is crucial for significantly enhancing patient outcomes and enabling dialysis care in Albania to approach global standards.

**Main Points**

- Malnutrition and protein-energy wasting are highly prevalent in hemodialysis patients.
- Socioeconomic barriers and cultural diets hinder effective nutritional management.
- In Albania, structured counseling, routine monitoring tools (such as bioimpedance spectroscopy), and culturally adapted education programs are largely absent, underscoring the need for system-level improvement.

## Introduction

Hemodialysis is a treatment option for patients with renal failure or end-stage renal disease (ESRD). It is performed by a machine that filters a patient’s blood outside the body. The patient’s blood is drawn through a tube in a dialysis machine, which cleans the blood and transfers it back to the body via another tube.^1^Conventional hemodialysis remains the most common treatment for ESRD worldwide and is usually performed for 3-5 hours, 3 days per week,^2^though alternative and intensified regimens are also employed for specific clinical indications. These include more frequent schedules (e.g., 4-6 sessions per week), short daily hemodialysis, and prolonged or nocturnal hemodialysis.^3^ Various risks are associated with hemodialysis. Patients may also experience acute or chronic complications. These include cardiac arrhythmias, hypertension, air embolism, bleeding, seizures,^4^ and autonomic dysfunction, characterized by reduced heart rate variability.^5^Beyond these complications, patients on maintenance hemodialysis often present with vascular calcifications, particularly in the aorta, and increased pulse wave velocity.^6^These conditions are recognized as significant risk factors for cardiovascular morbidity and increased mortality in this population.^7^ Chronic kidney disease and the dialysis process itself appear to accelerate arterial stiffening and vascular calcification, contributing to a significantly higher cardiovascular mortality risk in hemodialysis patients compared to the general population.^8^ Additionally, advanced kidney disease and renal replacement therapy lead to several metabolic and nutritional imbalances. Nutritional concerns in patients with ESRD undergoing maintenance hemodialysis are among the most highly valued in clinical management.^9^ Nutrition plays a major role in reducing complications and improving the quality of life; therefore, nutritional intervention should be an intrinsic part of the treatment modalities for such patients. These challenges include malnutrition, protein-energy wasting (PEW), hyperkalemia, hyperphosphatemia, and fluid overload.^10^ In Albania, there are 13 dialysis centers with 1607 patients. The annual incidence of new cases ranges from 300 to 400 cases per year. In recent years, there has been a focus on infrastructural, technological, and human resource investments. Despite the international guidelines available, their application in practice is not always uniform and mirrors a lack of resources.^10^

## Material and Methods

This article is a narrative review that synthesizes published evidence and contextual information on nutrition and fluid management in adults undergoing maintenance hemodialysis, with a particular focus on Albania and comparable low- and middle-income settings.

A broad, non-systematic search of the literature was conducted in PubMed, the Cochrane Library, Medscape, UpToDate, and Google Scholar to identify clinically relevant articles and guidelines published between 2000 and 2025. The search combined free-text terms such as “hemodialysis,” “nutrition,” “malnutrition,” “dietary management,” “fluid balance,” “micronutrients,” “protein-energy wasting,” “Albania,” “developing countries,” and “vitamin deficiency.” Both English- and Albanian-language publications were considered.

In addition, official websites of national health institutions such as Albanian Ministry of Health and Albanian Health Care Insurance Fundwere accessed to gather data and legislation relevant to hemodialysis care.^11^^,^^12^

## Results

### Nutritional Challenges

#### Vitamin and Mineral Deficiencies

International studies show that patients undergoing maintenance hemodialysis frequently present with multiple vitamin and mineral deficiencies that stem from reduced dietary intake, dialysis-related losses, and altered metabolism. Deficiencies in water-soluble vitamins are especially common: thiamine (B_1_) is often low enough to warrant supplementation, while vitamin B_6_, folate (B_9_), and B_12_ contribute to hyperhomocysteinemia and are frequently insufficient, and vitamin C deficiency is prevalent in patients not receiving supplements.^13^ Fat-soluble vitamin disturbances also occur: vitamin D deficiency affects 70%-80% of chronic kidney disease patients and is linked to secondary hyperparathyroidism, bone disease, and increased mortality, whereas vitamin A levels tend to be elevated despite reduced intake, reflecting impaired catabolism.^14^ Subclinical vitamin K deficiency is observed in a substantial proportion of hemodialysis patients, as indicated by low phylloquinone and high undercarboxylated osteocalcin or PIVKA-II levels.^15^ Mineral metabolism is similarly disrupted, with hypocalcemia, hyperphosphatemia, and elevated parathyroid hormone levels common in dialysis cohorts, especially among black patients who exhibit lower 25-hydroxyvitamin D and higher calcium, phosphorus, and Parathyroid hormone (PTH) levels.^16^ Overall, roughly half of the screened dialysis patients have at least 1 micronutrient deficiency, prompting KDOQI and other guidelines to recommend routine assessment and individualized supplementation of water-soluble vitamins and careful management of calcium-phosphate balance.^17^ To date, there are no published data on the prevalence of individual vitamin or mineral deficiencies among Albanian hemodialysis patients. Therefore, the patterns and proportions described above should be interpreted as global context rather than as confirmed Albanian epidemiology; they are used here to highlight potential risks that are likely relevant in Albania given similar dialysis practices and dietary constraints.

Carnitine deficiency is a universal problem in hemodialysis patients, with prevalence rates ranging from 25% for serum free-carnitine <20 µmol/L to >90% when considering the combined acyl-carnitine/free carnitine ratio and total carnitine loss during treatment.^18^ This deficiency arises because the kidneys are a major site of endogenous synthesis and reabsorption, both of which are eliminated by dialysis, and dietary intake is often restricted by protein-restricted diets. Clinically, low carnitine levels contribute to erythropoiesis-stimulating agent (ESA)-resistant anemia, intradialytic hypotension, muscle weakness, and cardiac dysfunction, all of which impair quality of life and increase morbidity. Current practice guidelines recommend levocarnitine supplementation for patients with ESA-resistant anemia, symptomatic hypotension, or cardiomyopathy.^19^

Oral regimens that achieve >4200 mg/week for at least 12 weeks have been shown to reduce dialysis-related hypotension and muscle cramps.^20^ Intravenous protocols typically use 1 g of levocarnitine administered post-dialysis 3 times per week, which improves hemoglobin levels, lowers ESA dose requirements, and alleviates muscle fatigue in most responders.^21^ Levocarnitine supplementation is not included in the state reimbursement dialysis treatment protocol in Albania, according to the current Albanian Health Care Insurance Fund.^22^

#### Malnutrition and Protein-Energy Wasting

A number of cross-sectional studies have demonstrated that malnutrition is a common problem in maintenance hemodialysis patients, with a prevalence of 43%-61%. For example, in Mauritius, malnutrition was described in 43.2% of patients,^23^ and in Tanzania, 61.2% of hemodialysis patients were malnourished, with 1.9% having severe malnutrition.^24^

The prevalence of malnutrition among hospitalized patients with advanced ESRD who were on hemodialysis was lower in another study (11.1%).^25^ Two studies in Palestine emphasized this burden; 1 study reported that 45.4% of patients were at high risk of malnutrition by renal iNUT, and another study found a prevalence of 28.2% using the Malnutrition-Inflammation Score. Likewise, in a study conducted in Iran, more than half (60.5%) were either mildly or moderately malnourished, and only 1% were severely malnourished.^26^ Another study from Spain found a national malnutrition prevalence of 51.7% in hemodialysis patients with moderate/severe malnutrition.^27^ Together, these international studies indicate variability in malnutrition prevalence across settings. In Albania, no published national prevalence data on malnutrition or PEW in hemodialysis patients are available; therefore, local burden is inferred from international studies.

The etiologies of PEW are attributed to poor energy or protein intake, concomitant chronic kidney disease, intercurrent acute illnesses, and perhaps enhanced production of inflammatory cytokines. Other factors include catabolic stimuli of hemodialysis, loss of nutrients to dialysate, primarily amino acids, peptides, glucose (when the dialysate is glucose-free), water-soluble vitamins, and diagnostic or therapeutic procedures, such as prednisone therapy. Other causes of PEW include continuous blood loss, anemia, and endocrine defects such as severe insulin resistance, resistance to insulin-like growth factor 1, hyperglucagonemia, hyperparathyroidism, and 1,25-dihydroxycholecalciferol deficiency. Metabolic products accumulating in renal failure may also contribute to the induction of potentially toxic substances such as aluminum.^28^ Recent evidence has highlighted the role of inflammation and changes in hormone regulation during the development of PEW. High levels of pro-inflammatory cytokines, such as interleukin 6 and tumor necrosis factor-α, cause increased muscle breakdown and lower appetite. Hormonal problems, including insulin and growth hormone resistance, worsen protein breakdown.^29^ The interaction between chronic inflammation, oxidative stress, and uremic toxins has been shown to accelerate muscle wasting regardless of dietary intake. This view suggests that PEW is not only the result of poor nutrition; it is also a systemic metabolic inflammatory disorder linked to ESRD.^30^

The diagnostic criteria for PEW include serum chemistry, such as albumin, prealbumin, cholesterol, body mass, muscle mass, and dietary intake.^31^ Patients undergoing hemodialysis face a high risk of developing PEW. This condition can lead to malnutrition, reduced physical function, and high rates of illness and death. While protein restriction is a common method in Chronic Kidney Disease (CKD) to lessen the load on the kidneys, patients on hemodialysis may require more protein due to losses during dialysis. This increases the chance of not getting enough protein.^32^

Hypoalbuminemia is a key marker for malnutrition. It is often overlooked despite being a strong predictor of poor outcomes.^33^ Body mass and muscle mass were not frequently considered.^32^ Research shows that the risk of malnutrition is linked to marital status (unmarried), higher body mass index (BMI), male sex, and lower levels of education.^34^ Available reports from Albania suggest limited nutrition assessment and follow-up in dialysis patients, particularly among rural populations. Most patients could not track their dietary intake, as many of them came from rural areas, while 47.7% came from urban areas. In addition, 87.1% of the patients had completed only elementary, middle, or high school, while only 9.3% had a university degree.^35^

#### Barriers to Adequate Nutrition

Nutritional challenges in Albania appear to be compounded by several systemic and cultural barriers. Economic factors also play a significant role. The World Bank classifies Albania as an upper-middle-income country,^36^ but 42.1% of patients live in low-income households. Only 50.7% belonged to the moderate-income category. Of these, 7.2% lived in high-income households.^37^ Therefore, many patients are unable to afford renal-specific food products or protein supplements.

Nutritional management is complicated by dietary habits. Traditional Albanian cuisine includes high amounts of phosphorus from dairy products and potassium from vegetables, creating diets that are challenging for patients. Albanians are among the largest consumers of milk and its products worldwide.^38^

Another challenge is the health infrastructure in Albania. Dialysis centers lack dietitians specializing in renal nutrition; thus, patients receive general advice from medical staff. A national audit report from the Albanian State Supreme Audit showed that no dietitians were found in Albanian hemodialysis units.^38^

A 2019 study conducted in Albania pointed out that 50% of the respondents stated that they had been educated consistently about the importance of a nutritional diet. However, adherence to this diet remains low.^39^ A significant challenge involves the engagement and understanding of patients’ relatives in the management of renal patients as it affects patient outcomes.^40^ In Albania, there are currently no official institutional initiatives specifically aimed at educating families of dialysis patients on disease management and nutritional support. Some health care institutions provide educational information on their official websites for patients and their families.^41^ Medical staff members are also reported to be involved in patient education. However, no published studies have assessed the level of education provided to patients and their relatives regarding nutritional management. Consequently, much of the discussion on educational barriers is based on limited local data and clinical experience, rather than on systematically collected outcome studies.

#### Fluid Management

Chronic fluid retention between dialysis sessions, caused by disrupted sodium and water balance, leads to interdialytic weight gain (IDWG) and results in excess fluid accumulation in the interstitial space. Excess IDWG predisposes many patients to comorbid factors associated with hypertension, heart failure, and intradialytic hypotension, accelerating the risk of cardiovascular mortality complications.^42^ In Albania, fluid overload is exacerbated by a lack of education and counseling programs. One of the most challenging aspects is adherence to fluid restrictions. Many patients describe thirst as a problem, especially with high-sodium diets or medications that enhance thirst, such as antihypertensives.^43^ This is compounded by the cultural norms of consuming salty foods in Albania. Albania ranks eleventh in the world for daily salt consumption per person.^44^ The majority of patients attending hemodialysis centers were reported to be between 51-70 years old.^39^ Poor awareness of the complications of fluid overload, particularly in elderly patients, leads to noncompliance, which is a factor in frequent hospital admissions and an increased risk of mortality.^45^However, reliable national mortality data specific to the Albanian hemodialysis population are currently unavailable in the published literature. In broader European cohorts, cardiovascular mortality remains the leading cause of death among hemodialysis patients, underscoring the clinical relevance of optimizing nutritional and fluid management even in the absence of country-specific survival statistics.^46^In this review, European mortality data are therefore used to frame the potential consequences of suboptimal fluid control in Albania, with the explicit caveat that they are extrapolated from other health systems.

## Discussion

Nutritional and fluid management are considered by the Kidney Disease: Improving Global Outcomes (KDIGO) and Kidney Disease Outcomes Quality Initiative(KDOQI) guidelines as important aspects of effective hemodialysis treatment. These factors have a direct impact on morbidity, mortality, and quality of life.^10^ However, there are some challenges to their implementation in Albania. There is no availability of dietitians, renal-specific food is not available, and there is a strong cultural influence on food choice. Patient education occurs sporadically in terms of frequency and quality, which reduces dietary and fluid restriction compliance.

Recommendations demand regular assessment of nutritional status. These assessments need to utilize a mixture of biochemical markers such as serum prealbumin and albumin, anthropometric measurements such as BMI, mid-upper arm circumference, and evaluation of dietary intake on a 3-month basis.^10^ This is particularly necessary in low-resource settings, where malnutrition and PEW are often undetected. Albanian dialysis units can do so through regular screening protocols integrated into dialysis regimens.

Fluid management should involve monitoring of IDWG and adjustment of dry weight based on objective evidence. Small increases in IDWG, greater than 5.7% of body weight, are linked to increased cardiovascular mortality.^47^ The best practice is to limit the net daily gain in fluid to a maximum of 1 L per day through sodium restriction. This needs to be complemented by formal patient counseling and written information in the patient’s language.^48^ Tailored counseling, especially during dialysis, allows the exploration of patient-level cultural and psychological barriers. Research has proven that this approach significantly improves target achievement.^49^

The KDIGO suggests that every dialysis unit has a specialized renal dietitian. Albanian facilities without such experts are at a disadvantage. Dietitians can create individualized, evidence-based meal plans within protein and energy targets per day, together with restrictions on phosphorus, potassium, and fluids. Cost is a barrier, but partnerships with local distributors, NGOs, and government initiatives could subsidize kidney-friendly foods and renal-specific supplements.

Guidelines internationally recommend the use of advanced monitoring tools, such as bioimpedance spectroscopy, to assess the extracellular volume status more accurately when possible. Limited resources may make it inadvisable to implement this widely in Albania. However, it is economical to reserve its application for repeated intradialytic hypotension, uncontrolled blood pressure, and persistent fluid overload.

Routine screening protocols should integrate comprehensive cardiovascular risk assessments, such as monitoring for arterial stiffness and autonomic health, alongside standardized nutritional and fluid status evaluations.^50^

Currently, there is no published Albanian report on malnutrition prevalence, dietary adherence, or IDWG trends in patients with ESRD. This absence should be a priority for research to be completed. Future multicenter studies can compare actual practice with guideline recommendations and measure the impact of formal interventions. These can include culturally tailored nutrition education, renal-specific supplements administered, and levocarnitine therapy in selected patients. Such evidence would merit the establishment of country-specific protocols that are possible within the resource limitations of Albania, but simultaneously use world best practices. Until such data are available, much of the present discussion necessarily extrapolates from international literature and should be interpreted as hypothesis-generating for the Albanian context.

## Conclusion

This review highlights the nutritional and fluid challenges faced by patients undergoing hemodialysis while drawing attention to the situation in Albania. Problems such as PEW, deficiencies of certain micronutrients, and poor fluid control are not only frequent, but also directly linked to worse outcomes.

The lack of standardized screening in Albania (such as prealbumin measurement, anthropometrics, and dietary intake) makes this problem largely invisible. Protein-energy wasting often goes untreated until it manifests as a severe clinical complication. Nutritional care in Albania is restricted not only by the healthcare system but also by cultural dietary norms. Traditional Albanian diets are high in salt, dairy (phosphorus load), and potassium-rich vegetables, which are particularly challenging for dialysis patients. Combined with low household income (> 40% of patients in low-income groups), this problem becomes more challenging.

Bridging this gap requires structured programs, such as the presence of renal dietitians, use of visual tools for tracking fluid intake and bioimpedance spectroscopy where feasible, culturally tailored education for patients and their relatives, partnership with local distributors, NGOs, and government initiatives for making affordable foods and renal-specific supplements.

Albania has no large-scale malnutrition prevalence, PEW, or fluid overload studies in the dialysis population. In the absence of these data, it is uncertain whether the global KDIGO or KDOQI guidelines can be adopted simply or need to be reworked for this environment. Multicenter collaborative research could yield such data and a stronger foundation for national policy and institutionalized nutritional regimens, making dialysis care approach global standards and improving survival along with patients' quality of life.

### Strengths and Limitations

This review has several strengths, including being the first to comment on nutritional and fluid management problems in Albanian hemodialysis patients; providing a useful comparison between international KDIGO/KDOQI guidelines and local practice; and taking an expansive view that incorporates biomedical, socioeconomic, and cultural barriers. Importantly, it moves beyond descriptions to offer practical recommendations for improving patient care. However, this study is limited by the absence of original Albanian prevalence data and reliance on findings from diverse international contexts. Additionally, the absence of publicly available national mortality statistics limits the ability to contextualize nutritional and fluid management challenges within survival outcomes.

### Plain Language Summary

Patients on hemodialysis often struggle with nutrition and fluid management, which can affect their health and quality of life. Many have PEW, low levels of important vitamins and minerals, and problems with fluid overload. In Albania, these issues are made worse by traditional diets high in salt, phosphorus, and potassium, low income, and a lack of dietitians in dialysis centers. Patients and their families often get limited guidance on how to eat or manage fluids properly. To improve outcomes, regular nutrition checks, simple diet plans, education, and better access to kidney-friendly foods and supplements are needed, along with local research to guide care.

## Data Availability

The data that support the findings of this study are available on request from the corresponding author.
